# The interplay of GTP-binding protein AGB1 with ER stress sensors IRE1a and IRE1b modulates Arabidopsis unfolded protein response and bacterial immunity

**DOI:** 10.1080/15592324.2021.2018857

**Published:** 2021-12-30

**Authors:** Taiaba Afrin, Caitlin N. Costello, Amber N. Monella, Camilla J. Kørner, Karolina M. Pajerowska-Mukhtar

**Affiliations:** Department of Biology, University of Alabama at Birmingham, 1300 University Blvd, Birmingham, AL, USA

**Keywords:** Unfolded Protein Response, Inositol-Requiring Enzyme 1, GTP-binding Protein B1, Pseudomonas syringae pv. tomato DC3000, Arabidopsis thaliana

## Abstract

In eukaryotic cells, the accumulation of unfolded or misfolded proteins in the endoplasmic reticulum (ER) results in ER stress that induces a cascade of reactions called the unfolded protein response (UPR). In Arabidopsis, the most conserved UPR sensor, Inositol-requiring enzyme 1 (IRE1), responds to both abiotic- and biotic-induced ER stress. Guanine nucleotide-binding proteins (G proteins) constitute another universal and conserved family of signal transducers that have been extensively investigated due to their ubiquitous presence and diverse nature of action. Arabidopsis GTP-binding protein β1 (AGB1) is the only G-protein β-subunit encoded by the Arabidopsis genome that is involved in numerous signaling pathways. Mounting evidence suggests the existence of a crosstalk between IRE1 and G protein signaling during ER stress. AGB1 has previously been shown to control a distinct UPR pathway independently of IRE1 when treated with an ER stress inducer tunicamycin. Our results obtained with combinatorial knockout mutants support the hypothesis that both IRE1 and AGB1 synergistically contribute to ER stress responses chemically induced by dithiothreitol (DTT) as well as to the immune responses against a phytopathogenic bacterium *Pseudomonas syringae* pv. tomato strain DC3000. Our study highlights the crosstalk between the plant UPR transducers under abiotic and biotic stress.

## Introduction

Eukaryotic cells rely on their plasma membrane-localized receptor proteins to sense the extracellular stimuli and send the signals to intracellular components.^1^ Among the receptor proteins, guanine nucleotide-binding proteins (G proteins) are universal signal transduction elements in all eukaryotes that have been extensively investigated due to their ubiquitous presence and diverse nature of action.^2^ The G proteins form typically plasma membrane-bound heterotrimeric complexes^3^ that function as hubs regulating responses to diverse developmental and environmental cues.^2,4–7^ The canonical G-protein complexes are composed of Gα, Gβ and Gγ subunits^8^ and mediate the action of seven transmembrane cell surface receptors known as G protein-coupled receptors.^2,8^ Typically, the plant genomes encode one Gα, one Gβ, and three to five Gγ subunits.^2^ For example, rice has one Gα, one Gβ and five Gγ subunits^9^ while Arabidopsis contains one Gα (AtGPA1),^10^ three extra-large Gα’s (XLG1/XLG2/XLG3),^11,12^ one Gβ (AGB1)^13^ and three Gγ (AGG1, AGG2, and AGG3) subunits.^14–16^

It is well established that the plant G proteins play important roles in a multitude of developmental responses to stimuli such as light, nutrients, sugar, and regulation of growth and stomatal density, among others.^2,8,17–22^ Moreover, G proteins are implicated in phytohormone signaling, most notably auxin,^18^ gibberellic acid,^23,24^ brassinosteroids (BR),^23^ abscisic acid (ABA),^25^ and jasmonic acid.^8^ Moreover, G proteins are also extensively involved in plant defense responses. Evidence suggests that AGB1 (Gβ), AGG1/AGG2 (Gγ), and XLG2/XLG3 (extra-large Gα) participate in Arabidopsis innate immune responses for defenses against a broad spectrum of pathogens.^8,26–29^ Additional reports indicate that the G proteins also constitute an integral part of resistance mechanisms against necrotrophic fungal infections.^8,26^

G proteins are primarily associated with the plasma membrane; however, a fraction of the Arabidopsis Gβ subunit, GTP-binding protein β1 (AGB1), was detected in association with the ER membrane,^30^ providing an intriguing connection between G proteins and the ER signaling. The ER, as the largest membrane system of a eukaryotic cell, plays a central and integrative role in the coordination of cellular transport and signaling.^31^ The ER coordinates the essential cellular processes such as membrane protein synthesis, folding, post-translational modifications, and peptide delivery to target locations, ensuring the maintenance of proteostasis.^31,32^ Biotic and abiotic stress can disrupt these processes, leading to the accumulation of malfolded or unassembled proteins in the ER, forming toxic protein aggregates that cause subsequent ER stress.^33^ The onset of ER stress triggers several responses to restore cellular homeostasis. Among those, unfolded protein response (UPR) is a universal form of the ER stress signaling executed by Inositol-Requiring Enzyme 1 (IRE1) and aimed at correcting the aberrant ER conditions and protecting cellular viability.^34–37^ IRE1 is an evolutionarily conserved transmembrane sensor serine/threonine kinase equipped with an N-terminal ER-resident stress-sensing domain and a C-terminal endoribonuclease domain.^37,38^ Arabidopsis contains three IRE1 homologs: IRE1a, IRE1b, and IRE1c.^36,39^ IRE1a and IRE1b are the full-length homologs extensively involved in ER stress signaling in response to various biotic and abiotic stimuli^36,39–42^ and share considerable amino acid sequence similarity especially within their cytoplasmic tails.^43^ Whereas, IRE1c is a truncated variant,^39^ which lacks the ER-resident N-terminal domain and plays a crucial role in gametogenesis in the absence of IRE1b.^39^ Upon biotic or abiotic stress, transcription and translation rapidly intensify, which places a burden on the ER protein folding machinery. The luminal domain of IRE1 senses the accumulation of misfolded peptides, leading to IRE1 homo-oligomerization, trans-autophosphorylation, and culminates in the activation of unconventional splicing of its cognate mRNA substrate *bZIP60* to mediate downstream signal transduction.^42,44^

Mounting evidence suggests the existence of a crosstalk between IRE1 and G protein signaling during ER stress. AGB1 has been reported to be involved in UPR through a pathway parallel to IRE1,^30,40^ as evidenced by the heightened sensitivity to chemical ER stress, aggravated short-root phenotypes, and decreased expression of a suite of ER chaperones in the triple mutants *ire1a/ire1b/agb1* when compared to *ire1a/ire1b* or *agb1* alone.^40^ Another report further corroborated the *AGB1*’s involvement in the sensitivity to tunicamycin (Tm; a potent inhibitor of *N*-linked glycosylation) and ER chaperone expression.^30^ In addition to its role in the ER stress responses, AGB1 is also implicated in diverse developmental and physiological processes, and the *agb1* mutants display several related phenotypes, such as reduced hypocotyl lengths, shorter siliques,^2,18,45–47^ altered leaf and flower shape,^18,47^ enhanced cell division in roots and excess lateral roots,^18^ higher stomatal density^21^ and altered metal ion profiles.^48^ Furthermore, AGB1 was shown to physically interact with a group I bZIP protein (VIP1),^49^ which is involved in the regulation of extracellular osmolarity and turgor pressure. The loss of AGB1 function additionally caused altered abiotic stress responses, for example, increased drought tolerance,^22^ hypersensitivity to salt stress,^50^ enhanced programmed cell death,^,51^ altered responses to hormones, i.e., BR, ABA, and auxin, as well as altered sugar sensing.^23,45,52–56^

Several studies reported the involvement of AGB1 in plant immunity,^57^ demonstrating reduced reactive oxygen species accumulation upon microbial infection,^56,58,59^ hypersensitivity to fungal infections by *Alternaria brassicicola*,^3,8^ *Botrytis cinerea*,^26^ *Plectosphaerella cucumerina*,^26,60^ and *Fusarium oxysporum*.^8,26,28^ An earlier report also indicated that AGB1 is involved in defenses against hemibiotrophic bacteria *Pseudomonas syringae*^59^ in a manner that is independent of salicylic acid (SA) signaling.

Here, we set out to provide more insights into the relationship of AGB1 and IRE1 in ER stress signaling and the mechanisms of resistance to *P. syringae*. We employed a genetic approach using single and combinatorial loss-of-function mutants of AGB1, IRE1a, and IRE1b to assess the differential sensitivity of these genotypes to two established ER stress-inducing chemicals, tunicamycin (Tm) and dithiothreitol (DTT), by measuring plant fresh weight and root elongation rates following chemical exposure. We also quantified the levels of susceptibility to infection with a phytopathogenic bacterium *P. syringae* pv. *tomato* strain DC3000. Our results showed that Arabidopsis AGB1 is required for effective ER stress and immune responses, and provided evidence suggesting that AGB1 works in parallel and synergistically with the IRE1 pathway to regulate ER homeostasis.

## Materials and methods

### Plant materials

**Figure 1.**
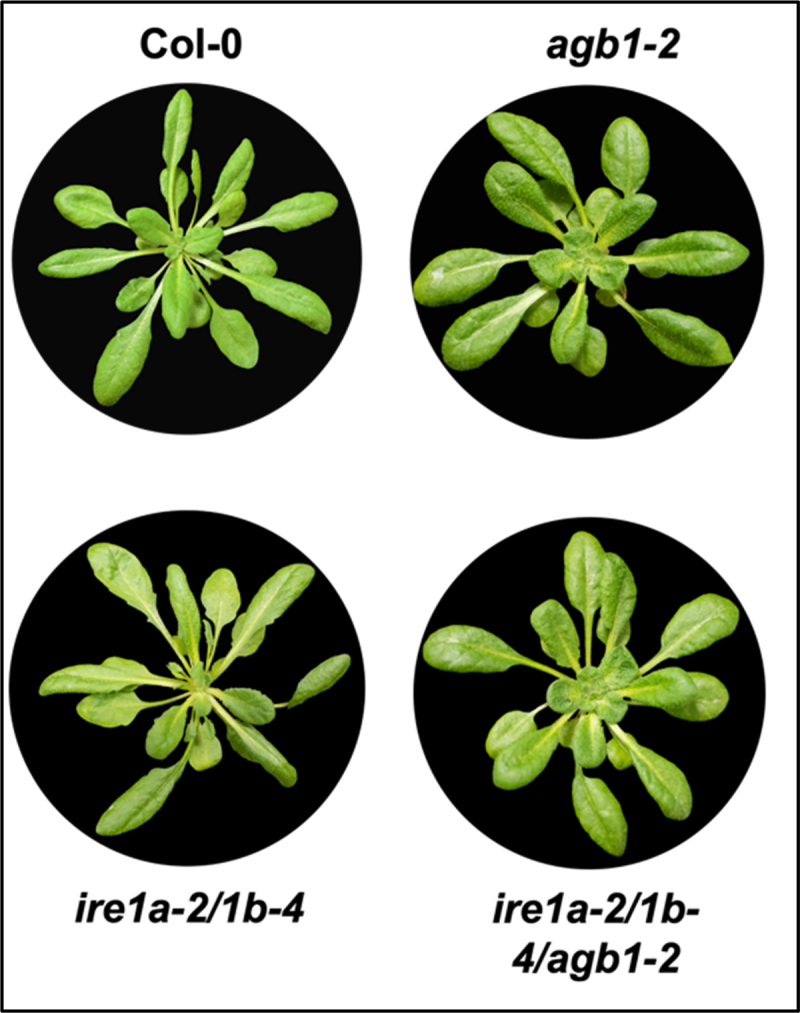
Representative phenotypes of Arabidopsis plants used in the study. Plants were photographed by NIKON D5600 camera. Images were prepared using Adobe Photoshop (Version: 21.2.4).

**Figure 2.**
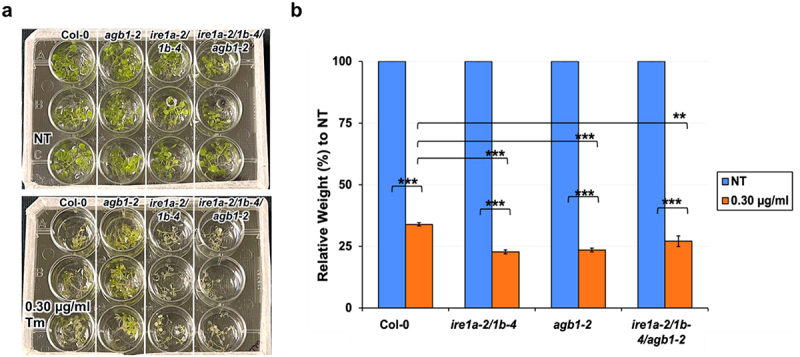
Analysis of chemical ER stress sensitivity to 0.3 μg/ml tunicamycin (Tm) on fresh weight of indicated genotypes. Seedlings were grown vertically on solid MS media for 7 days, then transferred to fresh liquid MS media without (NT = no treatment) or with Tm. Five days following Tm exposure. the seedlings were photographed (a) and total fresh weight of at least 30 plants per biological replication was recorded (b). At least three biological replications were performed. Statistical analyses were performed by two-tailed Student’s t-test or one-way ANOVA in Excel. Error bars show mean ± SD (n ≥ 30). Significant differences are indicated by asterisks (*** *p* < .001, ** *p* < .01).  Short solid bars connecting bars represent the comparison of fresh weight between untreated and treated samples for each genotype, while long solid lines represent the comparison of fresh weights of Tm-treated plants between Col-0 and an indicated mutant.

**Figure 3.**
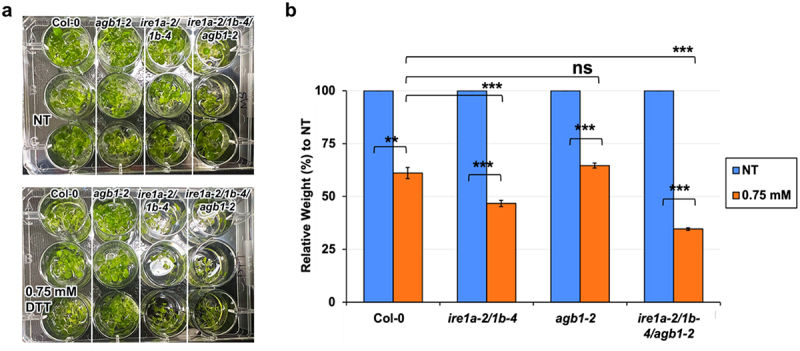
Analysis of chemical ER stress sensitivity to 0.75 mM DTT on fresh weight of indicated genotypes. Seedlings were grown vertically on solid MS media for 7 days, then transferred to fresh liquid MS media without (NT = no treatment) or with DTT. Seven days following DTT exposure, the seedlings were photographed (a) and total fresh weight of at least 30 plants per biological replication was recorded (b). At least three biological replications were performed. Statistical analyses were performed by two-tailed Student’s t-test or one-way ANOVA in Excel. Error bars show mean ± SD (n ≥ 30). Significant differences are indicated by asterisks (*** *p* < .001, ** *p* < .01), while “ns” indicates no statistically significant differences. Short solid bars connecting bars represent the comparison of fresh weight between untreated and treated samples for each genotype, while long solid lines represent the comparison of fresh weights of DTT-treated plants between Col-0 and an indicated mutant.

**Figure 4.**
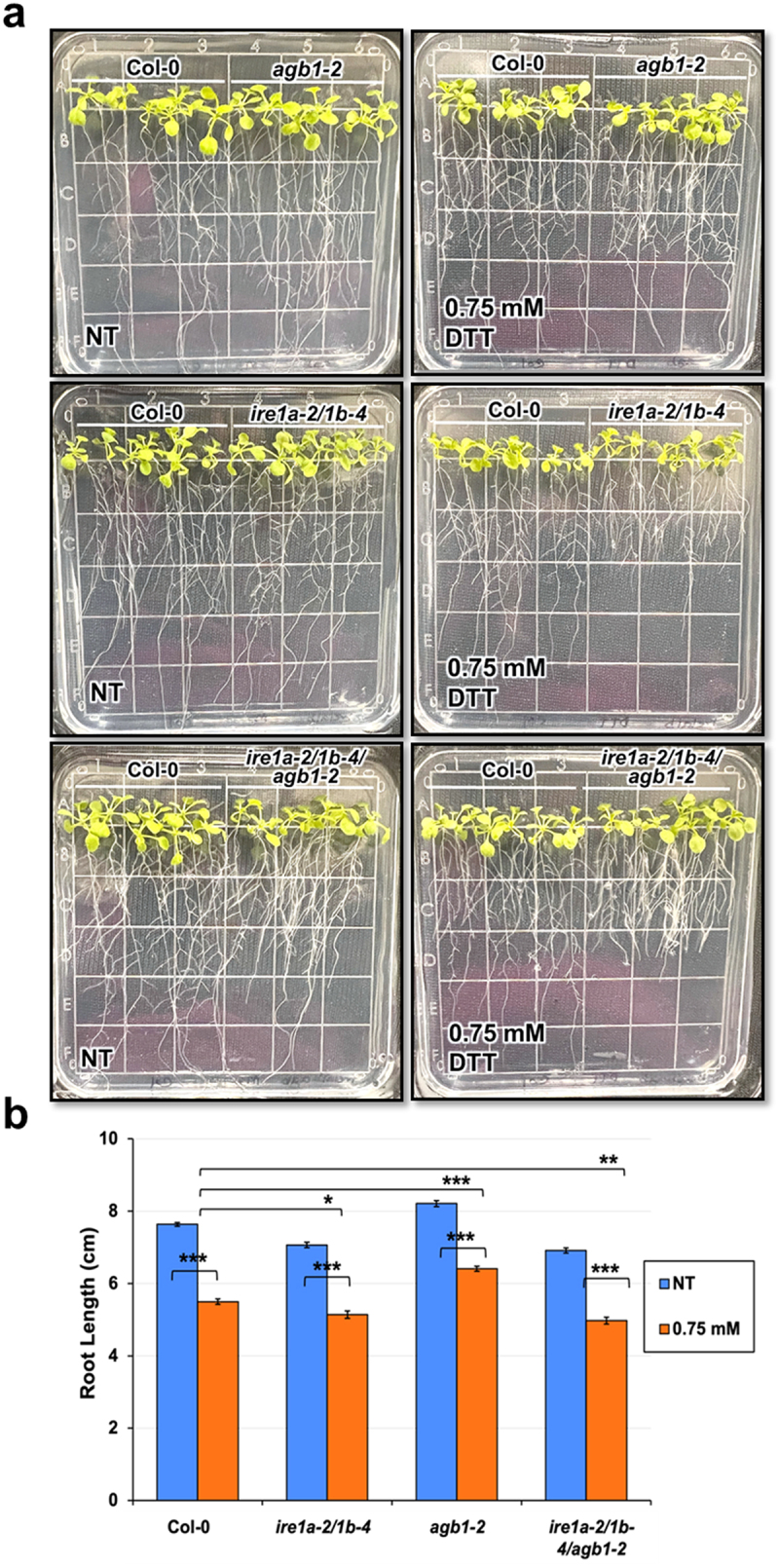
Analysis of root length in response to a chemical ER stress triggered by exposure to 0.75 mM DTT of indicated genotypes. Seedlings were grown vertically on solid MS media for 7 days, then transferred to fresh plates containing solid MS media without (NT = no treatment) or with DTT. After 7 days, the seedlings were photographed (a) and root length was measured using a ruler (b). An average of 15 seedlings was used per biological replication and at least four biological replications were performed. Statistical analyses were performed by two-tailed Student’s t-test or one-way ANOVA in Excel. Error bars show mean ± SD (n ≥ 30). Significant differences are indicated by asterisks (*** *p* < .001, ** *p* < .01, * *p* < .05). Short solid bars connecting bars represent the comparison of root length between untreated and treated samples for each genotype, while long solid lines represent the comparison of root length of DTT-treated plants between Col-0 and an indicated mutant genotype.

Ecotype Columbia (Col-0) was used as the control genotype in this study. T-DNA and EMS mutant lines *agb1-2* (CS6535), *ire1a-2* (SALK_018112), *ire1b-4* (SAIL_238_F07)^42^ and *npr1-1* (CS3726)^61^ were obtained from Arabidopsis Biological Resource Center (ABRC). The phenotypes of rosette leaves in all genotypes are illustrated in Figure 1. Phenotypes of seedlings treated with tm and DTT are displayed in Figure 2a, 3a and 4a. All pictures were taken by NIKON D5600 camera and images were prepared using Adobe Photoshop (Version: 22.4.2).

### ER stress response assays

Arabidopsis seeds were sterilized with a wash buffer (70% Ethanol and 0.05% Triton) and stratified at 4°C for 3 days on half-strength solid Murashige Skoog (MS) media plates (Phytotechnology Labs, Overland Park, KS, USA). The MS plates were then transferred to a growth chamber under a 12 h light/12 h dark photoperiod; 40% relative humidity; 21°C and 100 μmol/m^2^/s light intensity. The plants were grown vertically for 7 days, followed by the appropriate chemical ER stress treatment.

For tunicamycin (Tm) sensitivity assays, 7 days old Arabidopsis seedlings were transferred to 12-well plates containing liquid half-strength MS media supplemented with Tm concentration of 0.3 μg/mL (Tocris Bioscience) or mock (DMSO). After 5 days of Tm exposure, the total fresh weight of seedlings was recorded. 15 seedlings were used per biological replication and at least three biological replications were performed.

For dithiothreitol (DTT) sensitivity assays, 7 days old Arabidopsis seedlings were transferred to 12-well plates containing liquid half-strength MS media supplemented with 0.75 mM of DTT (ACROS Organics) or mock (ddH_2_O). After 7 days of DTT exposure, the total fresh weight of seedlings was recorded. An average of 15 seedlings was used per biological replication and at least four biological replications were performed. For root length assays, 7 days old seedlings were transferred to half-strength solid MS media plates with or without 0.75 mM of DTT. After 7 days, the root length was measured using a ruler. An average of 15 seedlings was used per biological replication and at least four biological replications were performed.

### Bacterial strain and growth quantification

For bacterial quantification assays, seedlings were sown in individual pots on sterilized soil (SunGro Horticulture, Super-Fine Germinating Mix) and transferred to a cold room facility for stratification at 4°C for 7–10 days. After stratification, the pots were transferred to a controlled growth room facility with 12 h light/12 h dark photoperiod; 40% relative humidity; 21°C and 100 μmol/m^2^/s light intensity. 10–15 days old seedlings were transplanted into 72-well flats for growth. 3–4 weeks old rosette leaves were infiltrated with *Pseudomonas syringae* pv. *tomato* DC3000 (*Pst* DC3000) (OD_600_ = 0.0002) using needleless syringes and bacterial growth was quantified after 72 hours.^62^ 3 leaves per plant, 6 plants per biological replication, and at least three biological replications were performed.

### Statistical analyses

Statistical differences were calculated by two-tailed Student’s *t*-test or one-way ANOVA in Microsoft Excel. RStudio (ggplot2) was used to generate the graph in Figure 3 while MS Excel was used to make graphs in Figure 2b, 3b, and 4b Statistically significant differences are indicated with *p < .05, **p < .01, ***p < .001, or ****p < .0001.

## Results

### Responses to ER stress

A decade ago, the Arabidopsis AGB1 was proposed to operate in an ER stress-responsive pathway that is independent of and parallel to IRE1a/IRE1b.^40^ While previous studies reported somewhat conflicting findings on the specific role of AGB1 in ER stress, ranging from enhanced sensitivity to enhanced tolerance,^30,40,63^ here we set out to better understand the possible combinatory effects of AGB1 with IRE1 homologs when exposed to different chemical ER stressors. Toward this, we crossed the *agb1-2* mutants with the *ire1a-2/ire1b-4* double mutant plants (further referred to as *ire1a-2/1b-4)* to obtain the triple mutant *ire1a-2/ire1b-4/agb1-2* (further referred to as *ire1a-2/1b-4/agb1-2*). All of these mutants showed distinguishable morphology from wild-type Col-0 under our growth conditions (Figure 1) and the previously described rounder rosette leaves phenotype of the *agb1-2* plants was also detected under our growth conditions in the *ire1a-2/1b-4/agb1-2* plants.^22^ Next, we subjected MS-media grown Col-0, *agb1-2, ire1a-2/1b-4* and *ire1a-2/1b-4/agb1-2* seedlings to treatments with 0.3 µg/mL Tm, which we previously determined to be the ideal concentration for the detection of mild defects in the UPR tolerance,^64^ and we quantified their total weight after 5 days of exposure. We found that all of the tested genotypes were sensitive to Tm, as indicated by the statistically significant decrease in the relative weight (*P*-value < 0.00001) when compared to their respective mock-treated control groups (Table 1).

**Table 1.** P-values from independent sample (two-tailed) *t*-test for fresh weight data resulting from the Tm treatment experiments

Comparison of Tm-treated genotypes:	*p*-value
Col-0 to *ire1a-2/1b-4*	< 0.00001
Col-0 to *agb1-2*	< 0.00001
*Col-0 to ire1a-2/1b-4/ agb1-2*	0.009688
*ire1a-2/1b-4 to ire1a-2/1b-4/ agb1-2*	0.037884
*agb1-2 to ire1a-2/1b-4/ agb1-2*	0.14394

We observed that the Tm exposure reduced the weight of the *agb1-2* plants more dramatically than that of Col-0 (Figure 2 a,b), which is in agreement with the previous findings on this specific *agb1* mutant allele.^40,63^ We detected an enhanced Tm sensitivity in the *ire1a-2/1b-4* seedlings, which was expected given the pivotal roles of IRE1a and IRE1b in plant ER stress responses, and is also consistent with the earlier reports.^64,65^ The triple mutant *ire1a-2/1b-4/agb1-2* displayed a statistically significant reduction in the fresh weight; however, its Tm sensitivity was not further enhanced compared to the double mutant *ire1a-2/1b-4* seedlings (Figure 2 a,b).

To substantiate our findings with Tm and further test the genetic relationship between AGB1 and IRE1a/IRE1b pathways in Arabidopsis chemically-induced ER stress, we exposed the mutants to another ER stress-eliciting chemical, dithiothreitol (DTT), and measured their total fresh weight 7 days following the treatment. We found that all of the tested genotypes were sensitive to 0.75 mM DTT and displayed a statistically significant reduction in their fresh weights as compared to their respective mock-treated control groups (Table 2). In response to treatment, the average fresh weight of the *agb1-2* plants was not significantly different when compared to that of Col-0 (Figure 3 a,b). Whereas, *ire1a-2/1b-4* double mutants showed a significantly increased DTT sensitivity, weighing ~30% less than the Col-0 control plants. The DTT-treated triple mutants *ire1a-2/1b-4/agb1-2* displayed a further reduction in their fresh weight compared to all other tested genotypes, indicating a synergistic effect of the IRE1a/IRE1b and AGB1 pathways on the Arabidopsis sensitivity to DTT-triggered chemical ER stress (Figure 3 a,b).

**Table 2.** P-values from independent sample (two-tailed) *t*-test for fresh weight data resulting from the DTT treatment experiments

Comparison of DTT-treated genotypes:	*p*-value
Col-0 to *ire1a-2/1b-4*	0.000273
Col-0 to *agb1-2*	0.269186
*Col-0 to ire1a-2/1b-4/ agb1-2*	< 0.00001
*ire1a-2/1b-4 to ire1a-2/1b-4/ agb1-2*	< 0.00001
*agb1-2 to ire1a-2/1b-4/ agb1-2*	< 0.00001

Given that we were able to better observe the genetic interaction between IRE1a/IRE1b and AGB1 using the DTT-induced ER stress treatment, we further investigated the effect of this compound on the inhibition of root elongation. We grew the above-described genotypes vertically on plates supplemented with 0.75 mM DTT and we observed that all of the experimental plants showed marked sensitivity to DTT exposure as reflected by a statistically significant reduction in root length (Figure 4 a,b and Table 3).

**Table 3.** P-values from independent sample (two-tailed) *t*-test for root length data resulting from the DTT treatment experiments

Comparison of genotypes:	NT to DTT *p*-value	DTT to DTT *p*-value
Col-0	< 0.00001	-
*ire1a-2/1b-4*	< 0.00001	-
*agb1-2*	< 0.00001	-
*ire1a-2/1b-4/ agb1-2*	< 0.00001	-
Col-0 to *ire1a-2/1b-4*	-	0.025996
Col-0 to *agb1-2*	-	< 0.00001
*Col-0 to ire1a-2/1b-4/ agb1-2*	-	0.002349
*ire1a-2/1b-4 to ire1a-2/1b-4/ agb1-2*	-	0.217358
*agb1-2 to ire1a-2/1b-4/ agb1-2*	-	< 0.00001

When grown under control conditions, *agb1-2* roots grew slightly longer than wild-type, whereas *ire1a-2/1b-4* and *ire1a-2/1b-4/agb1-2* produced roots shorter than those of Col-0. These findings are consistent with a previous study on these genotypes.^40^ Upon exposure to DTT, the *agb1-2* roots displayed a reduction in length but were still longer than those of Col-0 seedlings. In agreement with the fresh weight DTT assay results, the seedlings of the double mutant *ire1a-2/1b-4* showed a significant DTT sensitivity and produced shorter roots compared to Col-0 (Figure 4 a,band Table 3). Moreover, we detected a slightly more pronounced DTT sensitivity in the triple mutant *ire1a-2/1b-4/agb1-2*, which was the genotype with the shortest roots following the treatment, despite having comparable root size to the double mutant *ire1a-2/1b-4* when grown in the absence of the chemical ER stress (Figure 4 a,b). This result further substantiates the notion that the DTT-induced chemical ER stress involves a synergistic effect of the IRE1a/IRE1b and AGB1 pathways in Arabidopsis.

### *Responses to bacterial infection with* Pst *DC3000*

**Figure 5.**
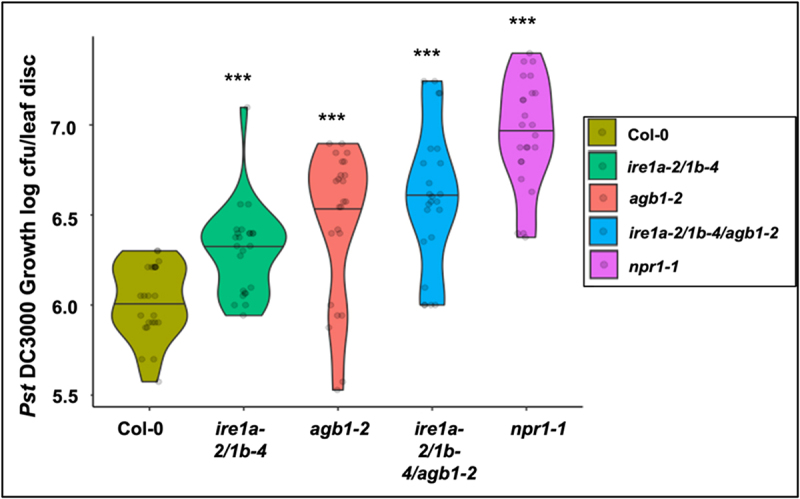
Bacterial infection with *Pseudomonas syringae pv*. tomato DC3000. Leaves of 4 weeks old plants of indicated genotypes were syringe infiltrated with the pathogen. *In planta* bacterial growth was quantified at 3 days post inoculation. The violin plots extend from 25^th^ to 75^th^ percentiles and whiskers extend from the minimum to the maximum levels. Light gray dots represent individual data points. Black lines in the middle represent the median. The data was generated from three independent biological replicates. Statistical analyses were performed in MS Excel by One-Way ANOVA. Significant differences in bacterial loads compared to Col-0 are indicated by asterisks (*** *p* < .001).

Earlier reports from our lab indicated that IRE1a and IRE1b play an important role in mediating the basal defense responses and systemic acquired resistance against *Pseudomonas syringae* infection.^42^ On the other hand, evidence exists in support of AGB1’s involvement in defense responses against *P. syringae,^59^* although the molecular mechanisms governing its contribution remain to be elucidated. Infection with *P. syringae* is known to cause an increased burden on the cellular translation, protein modifications, and secretion, which can lead to an overwhelmed ER function, accumulation of misfolded peptide aggregates and, in turn, severe ER stress.^66^ Given an indication that IRE1a/IRE1b operate in a signaling pathway independent of AGB1 during UPR signaling, as reported previously^40^ and inferred from the results of DTT sensitivity assays described above, we next asked if IRE1a/IRE1b and AGB1 have independent and possibly cumulative contributions to the immune response mounted against a virulent strain of *P. syringae Pst* DC3000. Toward this, we subjected the wild type Col-0 (positive control), *agb1-2, ire1a-2/1b-4* and *ire1a-2/1b-4/agb1-2* along with the hypersusceptible *npr1-1* mutant to *Pst* DC3000 infection. We used a low bacterial inoculum dose of *Pst* DC3000 (OD_600nm_ = 0.0002) to precisely assess the disease phenotypes in the individual genotypes. As expected, the Col-0 plants showed mild disease symptoms and limited pathogen proliferation (Figure 5), while the *npr1-1* exhibited the highest levels of bacterial accumulation, amassing ~31 times more bacterial colonies. The single mutant *agb1-2* and double mutant *ire1a-2/1b-4* displayed significantly enhanced bacterial loads compared to Col-0, which is consistent with earlier reports.^42,59,65^ The triple mutant *ire1a-2/1b-4/agb1-2* showed a further increased bacterial susceptibility compared to *ire1a-2/1b-4* and *agb1-2*, supporting 0.7 log (~5 times) more bacterial growth than Col-0 (Figure 5), and further substantiating the hypothesis that the IRE1 and AGB1 likely act non-redundantly and have a cumulative contribution to plant stress responses, including immunity to a bacterial pathogen.

Collectively, our results suggest that AGB1 contributes to both DTT-mediated chemical ER stress as well as pathogen-triggered ER stress in a manner that is distinct from and synergistic with the IRE1-mediated ER stress-responsive pathway in Arabidopsis.

## Discussion

The plant signaling pathways utilize a complex network of interactions to orchestrate biochemical and physiological responses in response to various stresses. To ensure adequate and integrated responses, plants often engage different signaling pathways that are interlinked with each other. In both animals and plants, G proteins have been well documented to act as hubs interconnecting various cellular signaling pathways.^67–69^ Our study showed that the Arabidopsis G protein subunit β1 (AGB1) cross-talks with the IRE1a and IRE1b homologs to modulate the abiotic and biotic ER stress response mechanisms. While the functions, mechanism of action, and importance of both IRE1a/IRE1b^34–36,40,42,65^ and AGB1^2,19,40,47,56,60^ in Arabidopsis have been well characterized, the nature of their cooperative roles in UPR remains unclear. Our findings support the notion that AGB1 and IRE1 signaling pathways are at least partially independent and can act synergistically in their response mechanisms, as proposed in an earlier study.^40^

Our study uncovered both commonalities and differences in how Tm and DTT engage AGB1 and IRE1a/b signaling pathways. This finding is not surprising given the distinct modes of action mediated by these two compounds. Tm causes ER stress by interrupting the enzyme GlcNac phosphotransferase, thereby preventing N-linked glycosylation.^70^ On the other hand, DTT is a strong reducing agent that inhibits disulfide bond formation during protein folding, which induces acute ER stress.^71^ Tm and DTT have been demonstrated to differentially affect the kinetics of ER stress and UPR target gene expression.^72^ In our study, seedlings treated with Tm showed enhanced sensitivity to this stressor, as illustrated by a statistically significant decrease in their fresh weights. While the *agb1-2* and *ire1a-2/1b-4* demonstrated heightened sensitivity, the combinatorial triple mutant *ire1a-2/1b-4/agb1-2* did not show further enhanced ER stress phenotypes, possibly because the conditions used by us have already maximized and saturated the responses mediated by the IRE1a/b pathway in the highly sensitized *ire1a-2/1b-4* mutant background. However, treatments with DTT exerted overall a milder degree of the ER stress than Tm and thus, provided a more sensitive experimental setup to detect the synergistic contributions of both pathways to ER stress responses, as demonstrated by the lowest fresh weights and shortest roots of the triple mutant *ire1a-2/1b-4/agb1-2* seedlings compared to *agb1-2* and *ire1a-2/1b-4.*

The specific dose and duration of the chemical ER stress treatment could be the reason behind some contrasting reports on the AGB1’s roles in ER stress. While earlier research using various Tm concentrations supported conclusions ranging from significant sensitivity of *agb1* plants^40,63^ to no substantial difference^40^ to enhanced resistance,^30^ the experimental setup varied between these studies, as did the age of seedlings, the concentration of Tm, duration of exposure to Tm, and the specific *agb1* T-DNA insertion mutant line used. Our conclusion is consistent with the findings of Chen and Brandizzi^40^ and Cho et al.,^63^ where the *agb1-2* plants were shown to have heightened Tm sensitivity. Moreover, our work provides additional experimental evidence for the role of AGB1 in chemical ER stress responses using a different stressor, DTT, and highlights the synergistic effects of IRE1a/b and AGB1 in this physiological process as previously proposed by Chen and Brandizzi.^40^ While the *agb1-2* plants did not show a marked reduction in their fresh weight and root length following DTT exposure, it should be noted that their fresh weights were higher and roots were longer than those of Col-0 under control conditions and we hypothesize that these phenotypes may give the *agb1-2* plants an advantage in withstanding the chemical ER stress. The effect of AGB1’s mutation, however, was clearly observed when the *agb1-2* plants were crossed into the highly sensitive *ire1a-2/1b-4* background. Hence, we concluded that AGB1 works synergistically with IRE1 during UPR induced by DTT to maintain the ER homeostasis.

Previous studies reported the independent contributions of IRE1a/IRE1b^35,36,42,65^ and AGB1^2,19,40,47,56,59,60^ to plant immune responses. In our study, we provide evidence that both IRE1a/IRE1b and AGB1 are required for initiating the basal defense response against the virulent bacterial pathogen (Figure 5), as the triple mutant *ire1a-2/1b-4/agb1-2* harbored a significantly higher number of bacteria than did the *ire1a-2/1b-4* and *agb1-2* plants. Under the infection conditions tested (inoculation with a low bacterial dose), the *agb1-2* plants showed a more susceptible phenotype than *ire1a-2/1b-4*, which indicates a trend opposite to the findings with DTT. This observation points toward an intriguing possibility that AGB1 may play a prominent role in the alleviation of biotic stress-induced UPR. Nonetheless, and consistent with the DTT results, the *ire1a-2/1b-4/agb1-2* triple mutants supported the highest levels of bacterial growth, further confirming the synergistic relationship of these two signaling pathways.

In summary, our study provided evidence of AGB1 contributions to both DTT-mediated chemical ER stress as well as pathogen-triggered ER stress in a manner that is distinct from and synergistic with the IRE1-mediated ER stress-responsive pathway in Arabidopsis. Our study highlights the novel aspects of crosstalk between the plant UPR transducers under abiotic and biotic stress.

## Data Availability

The authors confirm that the data supporting the findings of this study are available within the article or will be made available from the corresponding author, KPM, upon reasonable request https://authorservices.taylorandfrancis.com/data-sharing/share-your-data/data-availability-statements/.
